# Yoik experiences and possible positive health outcomes: an explorative pilot study

**DOI:** 10.1080/22423982.2016.1271590

**Published:** 2017-01-19

**Authors:** Soile Hämäläinen, Frauke Musial, Ola Graff, Torjer A. Olsen, Anita Salamonsen

**Affiliations:** ^a^ National Research Center in Complementary and Alternative Medicine, Departement of Community Medicine, Faculty of Health Sciences, UiT The Arctic University of Norway, Tromsø, Norway; ^b^ The University Museum of Tromsø, UiT The Arctic University of Norway, Tromsø, Norway; ^c^ Centre for Sami Studies (SESAM), Faculty of Humanities, Social Sciences and Education, UiT The Arctic University of Norway, Tromsø, Norway

**Keywords:** Culture-sensitive research, emotion management, health and well being, indigenous methodology, music and health, Norway, Sami, yoik experience, yoik

## Abstract

**Background:** Yoik is an old vocal music tradition of Sami, the indigenous people inhabiting Northern Fennoscandia and Kola peninsula in Russia. Studies of music therapy (MT) and especially singing have documented improvements in social and overall functioning in people with severe mental disorders and positive effect on depressive symptoms and sleep quality. Possible connections between yoik and health are so far underexplored.

**Objectives:** The overall aim of this study was to explore whether yoik may have the potential to positively influence people’s health and well-being. The research questions were: 1. What are different persons’ experiences with yoik? 2. Can yoik experiences be related to health outcomes?

**Methods:** Explorative, qualitative interviews with 13 participants were conducted in the Norwegian counties Finnmark, Troms, Nordland, and Trøndelag.

**Findings:** The findings suggest qualities in yoik that are comparable to positive effects of Music Therapy (MT) in general. Yoik may contribute to emotion management, i.e. processing negative emotions and inducing positive ones in people acknowledging yoik as something positive.

**Conclusion:** Yoik may be considered an important marker of social and cultural belonging for many Sami people. Yoik seems to have an underresearched potential as an intervention in culture sensitive healthcare and health promotion work that deserves to be further investigated.

## Background

The history of the Sami people of the circumpolar Fennoscandia is in many aspects the history of other circumpolar indigenous people, including colonisation, discrimination and marginalisation over centuries [1,2]. Important examples from Norway are Christian missionary prosecutions against Sami because of “sorcery”, i.e. practising pre-Christian religious rituals [3–5], ownership of land reserved for Norwegians only [6], and the period of “Norwegianisation” from about 1850 to 1959 [4,7]. During this period, the governmental aim was to shape the Sami into “good Norwegians” by suppressing Sami identity, denying the native language and replacing it with Norwegian language, and establishing Norwegian cultural markers. During this period, many Sami children were housed in boarding schools, away from their familiar cultural context [1,8–10].

Based on public health studies in other circumpolar first nations with a similar history, such as the Iñupiaq in Alaska and Kalaallit of Greenland, it is reasonable to assume that this history would influence Sami public health too. However, studies show that the Sami exhibit better overall health status, and do not suffer the same high rates of substance abuse, suicide, or unemployment [1,2,10]. Furthermore, the incidences of cardiovascular diseases, diabetes, lung cancer, and different infectious diseases among the Sami does not differ significantly from that of the majority of the Norwegian population. They are, however, remarkably better than respective rates in other circumpolar and arctic populations. It has been hypothesised that the early post-World War II governmental investments in an equal educational and healthcare system for all citizens have adjusted the socio-economic and public health differences between the Sami and the majority population (1,2,10). However, it is also possible that other significant factors have been influential, such as the rising of Sami self-awareness in the post-World War II era, culturally as well as politically [11,12,13]. So far, the possible positive influence of such cultural and social factors on health in Sami areas is largely underestimated. This article is thus based on a pilot study that explored a Sami cultural factor, the yoik, and its possible potential in health promotion in forms of everyday emotion management and self-expression.

### Yoik and its position among Sami people

Yoik is considered one of the oldest music forms in Scandinavia [14–16]. It allows and cherishes a wide range of vocal expressions, and melodic and rhythmic variations [5,9,15–17]. Yoik is comparable with singing because both are vocal expressions, but they differ in important aspects such as yoik’s referential symbolic function, i.e. that yoik refers to and symbolises an object/subject musically and thus, according to yoikers, always expresses something directly with its melodic and rhythmic organisation [3]. Yoik can be considered a very significant cultural means of communicating within Sami culture. In particular, the personal yoik communicates core values in the lives of many Sami, such as cultural identity, connection to a community, and the value and dignity of a person. There is nothing similar to the personal yoik in other Scandinavian music and singing traditions. It communicates deeply and directly a person’s place in and significance for his or her social surroundings [3,5].

Personal yoik is given away from one person to another, often from a parent/grandparent to a child as an expression of love and affiliation. The yoiker expresses his/her perception/impression of the character of the other person in the melodic and rhythmic organisation of the yoik, as well as in the vocal performance. The yoiker may or may not use a few words, such as the person’s name. A personal yoik is usually a positive acknowledgement of a person, a personal attribute that accompanies the person throughout life.^1^ ^1^However, yoik expressing negative feelings towards someone does exist. Graff [3] mentions a yoik made by a jealous young man to his rival. Yoiking your own yoik is considered as bragging. Thus, a personal yoik is also an important marker of social and cultural belonging; as long as someone is yoiking you, you know you belong to a community [3,9].

Yoik today is a part of the Sami cultural revitalising movement. At the same time that we have the first generations growing up without a personal yoik, the young Sami are embracing their cultural heritage in new forms, and yoik is taught and studied in kindergartens, schools, weekend workshops, and especially at festivals. Hilder even claims the festivals today being a kind of “indigenous museum” [12: 181] where long-lost archived yoik materials are coming to life again by being practised [12].

### Music therapy, health and emotion regulation

Music therapy (MT) may be defined as “the clinical and evidence-based use of music interventions to accomplish individualized goals within a therapeutic relationship by a credentialed professional who has completed an approved music therapy program” [18].

MT contains a wide range of methods from listening to recorded music, actively making music using instruments including your own voice, moving to music, and/or writing songs [18]. During the last decade, MT as a non-pharmacological intervention for several diseases has shown a variety of benefits. A systematic review documented improvements in social and overall functioning in people with severe mental disorders, as well as positive effect on depressive symptoms and sleep quality. Improvements in communication, better quality of life, and reduction in feeling isolated were reported. In cases with Parkinson’s disease, improved motoric functions related to the use of MT were revealed [19]. A literature review of effects of arts concluded that music reduced anxiety and stress, and enhanced relaxation [20]. The same was found for patients with coronary heart disease [21]. Studies have also revealed that singing may improve speech flow and intelligibility in neurological disorders such as Parkinson’s disease and aphasia [22,23]. MT studies have also documented the effectiveness of preferred and/or familiar music [24] and singing [25] in emotion regulation, i.e. reducing unwanted and inducing preferred emotions [24,26]. MT based on familiar music and/or songs can improve memory, communication, mobility, moods and behaviour, and reduce anxiety and depression in persons with unspecified dementia [27–30]. In late-stage Alzheimer’s disease, patients who were singing familiar songs improved their conversational abilities, mood, sense of belonging and worthiness [31]. We hypothesise that such positive connections between singing and health might also apply to yoik, because yoik is basically one traditional indigenous mode of singing.

Despite the positive results of studies of MT/familiar songs and the fact that yoik is an important marker of social and cultural belonging among Sami people, we have only found one single study that so far has investigated yoik in a healthcare context [32]. The focus was on successful intercultural communication in dementia care, and yoik served as an example of the significance of culture-specific symbols [32]. There is thus a lack of knowledge on yoik’s possible significance for positive health outcomes. This applies in general to indigenous singing traditions and possible connections to health. Ethnomusicology has mapped a lot of indigenous music and health traditions, but the connections are mostly investigated with regard to ceremonial and ritual practices, i.e. intentional healing practices [33,34]. The possible health benefits of everyday singing traditions of indigenous peoples seem to be rather unmapped, also within medical ethnomusicology, which is about “practices of music and healing […] from a cultural standpoint” [35].

### Aims and objectives

The overall aim of this study was to explore yoik’s possible significance for health in terms of well-being and quality of life. The research questions under investigation were:

1. What are different persons’ experiences with yoik?
2. Can yoik experiences be related to health outcomes?

## Participants and methods

### Participant recruitment and characteristics

Participants were recruited in several ways. The criteria for recruitment were (i) any experience of yoik, either performing or receptive, and (ii) willingness to talk about yoik experiences. According to Graff [3], yoik was originally a common type of musical expression and communication all over areas inhabited by Sami [3]. Today however, people seem to have different views on yoik: there are those who yoik and/or cherish yoik, and those who regard yoik as a sin and condemn it [3,9,15]. The reason for this might be found in the assimilation processes starting from Christianisation in the early 17th century, culminating in the period of Norwegianisation from 1851 to 1959 [4,7] and the complex aftermaths of these processes [36]. Possible positive and negative attitudes and emotions related to questions about yoik in participants were acknowledged in the recruitment process and the interviews.

The first author participated as part of the audience in events concerning the topic of yoik, and other Sami health or cultural matters. During these events, she asked nine persons that had spoken in public if they would be interested in participating in a pilot study investigating possible connections between yoik and health. They were told that the aim of this study was to explore whether a larger study on this issue was interesting and possible to conduct. Two other possible participants were contacted via Facebook. Furthermore, an e-mail request was sent to the leader of Juoigiid Searvi (the Yoiker’s Union), who forwarded it to the members. In addition, two persons regarded by the research group as key informants were approached by e-mail, and a third one by phone. All these possible participants then received information letters about the project. If they agreed to participate, they returned a declaration of consent. Six out of nine who were approached in arrangements consented. From the Yoiker’s Union three were interested, and one consented. One of three of the key informants consented. Finally, five participants joined the project through “the snowball sampling method” [37], where participants invited other possible participants in co-operation with the first author. Altogether, 32 possible participants were contacted and 26 letters of information were sent or handed out. Thirteen persons with an age span between 24 and 77, different levels of general and music education, professions, and various yoik backgrounds, gave their consent to participate (Table 1).

**Table 1.** Participant characteristics.

n=13	Women	Men
**Gender**	6	7
**Age**	58–77	24–70
**Sami**	5	6
**Non-sami**	1	1
**Yoik background** Grown up with yoik	5	3
**Education, not musical**		
<7 years	2	
7–9 years	1	
Higher education, 4 years or less	2	3
Higher education, more than 4 years		1
**Education, musical**		
High school		1
Higher education 4 years or less		1
Higher education Master’s level or more	1	1
**Musicians**		
Professional	1	3
Amateur		2
**Yoiking practice**		
Yoikers	5	5
Non-yoikers	1	2

The majority of participants were yoikers and Sami. The definition of a “yoiker” here means a person who has any experience of active yoiking by him/herself. Only a few were professional musicians, here defined as people who make their living primarily through playing one or more instrument(s). This also includes instrument teachers and music therapists, performing and/or composing music. The level of musical education varied. The definition of “Sami” is here based on what the participants spontaneously said about themselves, for example: “Although I’m Sami, yoik was considered a sin in my family…” and/or “Although I’m not Sami, I experience yoik as something extraordinary powerful…”. Therefore, the categories “Sami/not Sami” here express a subjectively experienced cultural identity [38]. The gender balance related to the total age span is unequal. This is due to the total amount of time available to conduct the study, combined with the persons available to be interviewed within a certain period of time. In other words, we did the interviews that were feasible. Of those who were invited but not participated, four persons directly refused, three because of “lack of knowledge and/or time” and one because of religious reasons. The rest either did not respond to written inquiries, or kindly expressed interest but without making commitment.

### Methodological approaches

The under-researched and experience-based issue under investigation demanded a flexible and open-ended research design, and thus a qualitative methodological approach was chosen. An explorative, qualitative approach was also suited to develop knowledge that could generate empirically and theoretically based hypotheses for a larger study [39]. We chose an inductive methodological approach, and based our empirical analysis on participants’ descriptions of, and reflections on, their yoik experiences. Although we had in mind the documented positive effect of music and singing in several therapeutic contexts, and hypothesised that yoik might have a similar function, we chose not to directly mention such a connection during the interviews if the participants did not introduce the connection themselves. Based on patterns revealed in the analysis of the conversations with the participants, relevant theories of emotion management and salutogenic and culturally sensitive healthcare were included.

### Qualitative interviews

Individual face-to-face interviews were conducted by the first author in places chosen by the participants. This included cafés, private homes and outdoors. The interview sessions varied from 1 to 3 h. The interviews were directed toward understanding the participants’ perspectives on their experiences as expressed in their own words [40]. We understand research interviews to be interactional, reciprocal, and reflexive processes in certain social and cultural contexts. Interview data are thus perceived as socially constructed, based on the interaction between researcher and participant [41–43].The main interview themes were: yoik experiences, the eventual prerequisites or contextuality of yoik experiences, and yoik compared with singing. A semi-structured interview guide was developed with input from all the authors in our multidisciplinary research group. The interview guide functioned as a checklist to ensure that every interview would include the same core topics. The interviews were conducted between March and May 2015, recorded digitally, and transcribed verbatim and anonymised by the first author. The interview languages were Finnish and Norwegian. When the first author/interviewer apologised for not being able to do the interviews in Sami, the Sami-speaking participants were rather surprised; none of them had even thought of that being an option.

### Content analysis

The interview transcripts were analysed through content analysis, i.e. the systematic classification process of coding and identifying different themes or patterns [44]. The interviews were first intensively read as a whole to gain a general understanding of different aspects of the material with relevance to the main research questions. The interviews were then re-read and coded in empirical and theoretical terms and the codes were discussed and confirmed in meetings between all authors.

### Ethics

Possible participants received letters of invitation that included ethical information and information about the study aim. Those who agreed to participate provided their written consent. Voluntary participation as well as the option of withdrawal at any time was emphasised both prior to and during the study. The authors have not provided any information in this article that may identify participants. The interviews were conducted with sensitivity to the situation of each of the participants [45] and with a culturally sensitive research approach [46]. As yoik might have very different connotations to different people, a culturally sensitive research approach in this project would mean awareness of this variety and awareness of possible consequences of participating in the study. For the first author who conducted the interviews, it also meant being aware of her possible lack of insight into sensitive issues that the research theme might generate. Thus, she ensured that the participants were the ones who would determine the amount and the type of information they would share, having empathic communication as her overall attitude [47,48]. The latter included also making the participants aware of the potential emotional responses from talking about a personal and possibly sensitive topic, and inviting them to contact her any time for any reason.

## Findings

### Yoik may function as emotion management

Ten out of 13 participants were yoikers. Eight had had yoik as part of their adolescence. The majority of the participants (nine out of 13) said that they were currently not exposed to yoik in their everyday lives.

The overall result derived from the empirical analysis was that there seem to be possible connections between yoik and positive health outcomes. Both yoikers and non-yoikers, being either Sami or Norwegian, reported feelings like “joyful”, “light”, “easy”, and “connected to something beyond myself”, when either yoiking or hearing yoik. Those who had personal yoiks felt “deeply honoured and acknowledged”, “seen as I really am” and “touched in my innermost being” when someone yoiked them. Those who yoiked actively, used words like “free”, “flow”, “intuitive”, “touch” and “deeper” when characterising their yoik experiences.

When the participants were asked about yoik settings – “When, where and what do you yoik?” – their answers revealed that yoik is used in both social situations and when being alone. Personal yoik is used to greet, lift up and honour a person, but also to recall a person not present: “When you think about someone or miss a person and you yoik that person, it is as if the person is there with you”. Several had experiences from personal yoik being used to process a loss of a loved one. One participant explained: “When a close relative died it was very difficult, but I kept on yoiking this person’s yoik… hour after hour… and gradually… I felt I came through the worst peak of the sorrow”.

Also landscapes, atmospheres, places, situations, weather, animals and emotions were yoiked: “When I’m really angry I yoik a special yoik so everybody can here it and know I’m angry!”, said one. Another said: “… we had walked all the long way up on the mountain, and there they were, turf after turf with these golden berries, and it was all so sweet, so the yoik just came out of me …”.

You know I’ve been seeing that mountain all my life, but first when I yoiked it I felt I got really close to it… like yes, it’s my, and my family’s and my ancestors’ mountain… we’ve wandered there for generations… it is ours, it is part of us… I felt a different kind of connection to it when I yoiked it…

When the participants were asked to summarise what yoik means to them in one word or a short sentence, many of them said: “Yoik is communication, a way of remembering”, “Yoik is like having a friend” and “Yoik is a connection to something beyond myself”. Some said it meant “Everything”. Many participants also meant that yoik is a culture-specific means of self-expression.

It is also noteworthy that those participants who had experienced hurtful restrictions to yoiking had kept on with it, or if they had stopped, they had later started yoiking again. One said: “Yoik has survived such a long time because it has had value for people. Otherwise it would have died out”.

## Discussion

The introductory presentation of MT research revealed various benefits of music and singing used as non-medical interventions [25–30]. The findings in this pilot study suggest qualities in yoik that are comparable with positive effects of MT in general. Yoik might have a function as what could be described as an ongoing musical self-regulation with a number of benefits following a continuous musical expression of one’s reality with the means always at hand – one’s own voice.

The empirical patterns derived in this study give a complex picture of the significance of yoik for the participants. Yoik’s significance is not bound to being a practising yoiker yourself. Nor is it bound to a person’s cultural or musical background, gender, age or general education, or to being continuously exposed to yoik. These findings imply something about the power of yoik in general for the participants in this study. We acknowledge also the particular significance and symbolic value of yoik for those of the Sami who appreciate yoik. The fact that yoik, despite the many restrictions, has survived through centuries and is still vividly alive and practised, indicates deeper meanings still to be explored.

### Yoik as emotion management

The common response of feeling “joyful”, “connected”, “honoured” and “happy”, despite different ages and backgrounds, indicates that yoik can have a capacity to lift people up. Also, yoik being described as “like having a friend”, and yoiking used to cope with painful or difficult emotions such as loss, anger and sadness indicate that yoik can have qualities and meanings that contribute to emotion management, i.e. how people relate to and handle their emotions (see *Emotion regulation, emotion management and self-regulation*).

When you have a friend you are not alone, you are supported. Being supported helps to keep your balance in life’s ups and downs, as does expressing and managing your emotions instead of suppressing them [49]. In our interpretation, yoik is being used as emotion management by the participants in this study. Yoik may be considered a tool to express and process negative emotions, as well as expressing and inducing positive emotions.

Emotion management is important because all emotions, both positive and negative, correspond to different physiological reactions. This is mostly known from studies of stress and the fight–flight response, but applies to other emotions as well [50,51]. According to Antonovsky, the human organism cannot keep a constant state of arousal due to emotional charge, no matter whether the emotions are pleasurable or unpleasurable [49]. Emotion management and self-expression seem to be necessary for our self-regulation competence, i.e. our ability to manage our emotions and behaviours, and take care of ourselves in different life circumstances. This is crucial for our salutogenesis, i.e. how we promote and maintain our health and well-being [49]. Our salutogenic ability – and thereby our self-regulation ability – in different, and especially, in challenging circumstances, is commonly called resilience [20]. Based on the participants’ descriptions of their experiences with yoik, it is reasonable to assume that yoik may contribute to such essential abilities for people who relate to it.

### Emotion regulation, emotion management and self-regulation

In this article, we have used the concepts “emotion regulation”, “emotion management” and “self-regulation”. The term “emotion regulation” is used in the MT literature when various types of MT have been implemented by healthcare professionals in order to regulate the emotions of the participants. “Emotion management” is a term we use to describe how the participants in this study use yoik as a self-administrated application in life in order to handle/manage their own emotions. We therefore perceive emotion management as a component in self-regulation. Based on this study, we argue that yoik seems to have the potential to contribute to emotion management and thereby self-regulation for those who include yoiking in their lives either by yoiking actively, listening to yoik, or being yoiked by someone. This may open up several possibilities in applying yoik in culturally sensitive healthcare practice and healthcare education [38,52,53]. Furthermore, it is reasonable to assume that using yoik as “familiar music” in adequate healthcare settings could be equally beneficial as in cases described in MT in general [24,25,27–30]. This could be the case, for example, for dementia care, psychological disorders such as depression and anxiety, or as part of rehabilitation procedures in neurological disorders such as Parkinson’s disease, aphasia or stuttering [22–30].

We have not succeeded in finding any studies about indigenous health and well-being considering the significance of singing traditions as such. The studies we have found refer to singing as part of traditional pre-Christian ritual or ceremony, which are then reported as contributing to health and well-being. Our approach to yoik was explorative; we wanted the participants to word their yoik experiences themselves despite the yoik context. The Sami yoik may, and may not, be used as a part of ritual and ceremony, both Christian and pre-Christian. The ritual use of yoik may potentially be a controversial topic among the Sami. However, to discuss the reasons for this is beyond the scope of this paper. Therefore, we chose to leave the yoik context open to the choice of the participants to describe if they wanted to. We found it sufficient if the participants shared their yoik experiences in their everyday, mundane lives.

### Methodological considerations

We do not claim to have gathered neutral, “objective” information on yoik experiences in this qualitative and explorative study. The interviews were particular social situations in particular contexts, where the participants related both to events and their meanings [43,54,55]. A possible weakness with qualitative research may be the lack of generalisability. However, the open-ended research questions in this study are not directly transferable to a quantitative design. Moreover, the aim of the study was not to make empirical generalisations the way empirical generalisation is possible in, for example, survey research, but to transfer experience-based knowledge as working hypotheses that can provide a valuable contribution to unexplored topics [39] such as yoik experiences and a possible positive connection between yoik and health. Because of time and resource limits, we were able to do only one set of interviews. Repeated interviews might have added more in-depth information [56]. Narrative analysis might have offered richness and additional dimensions [38].

This was a pilot study, however, and we argue that important empirical patterns that deserve further investigation have been revealed because of the chosen methodological approach. Through its illumination of people’s perspectives and experiences, qualitative research methodology may contribute a particular type of useful evidence for caring practices. In our opinion, qualitative study results such as those revealed in this study have the potential to be meaningfully translated into practice in ways that place people as human beings at the centre of care [57,58].

## Conclusion

Yoik does not necessarily have a conscious and outspoken purpose. To search for its function and potentially health-bringing potential implies looking for what is not necessarily said out loud. Still, based on the findings of this study, it seems that yoik may be good for people’s health as a means of emotion management and self-regulation. Yoik may give a sense of belonging for many Sami people. This study indicates that yoik may have an underresearched potential as an intervention in culturally sensitive healthcare and health promotion work that deserves to be acknowledged and further investigated.
